# A method for inferring medical diagnoses from patient similarities

**DOI:** 10.1186/1741-7015-11-194

**Published:** 2013-09-02

**Authors:** Assaf Gottlieb, Gideon Y Stein, Eytan Ruppin, Russ B Altman, Roded Sharan

**Affiliations:** 1 Departments of Bioengineering & Genetics, Stanford University, 318 Campus Drive, Stanford 94305, USA; 2 Sackler School of Medicine, Tel Aviv University, Klausner St., Tel Aviv 69978, Israel; 3 Department of Internal Medicine "B", Beilinson Hospital, Rabin Medical Center, 39 Jabotinski St., Petah-Tikva 49100, Israel; 4 Blavatnik School of Computer Science, Tel-Aviv University, Klausner St., Tel Aviv 69978, Israel

**Keywords:** Patient similarity, Electronic health records, Diagnosis prediction

## Abstract

**Background:**

Clinical decision support systems assist physicians in interpreting complex patient data. However, they typically operate on a per-patient basis and do not exploit the extensive latent medical knowledge in electronic health records (EHRs). The emergence of large EHR systems offers the opportunity to integrate population information actively into these tools.

**Methods:**

Here, we assess the ability of a large corpus of electronic records to predict individual discharge diagnoses. We present a method that exploits similarities between patients along multiple dimensions to predict the eventual discharge diagnoses.

**Results:**

Using demographic, initial blood and electrocardiography measurements, as well as medical history of hospitalized patients from two independent hospitals, we obtained high performance in cross-validation (area under the curve >0.88) and correctly predicted at least one diagnosis among the top ten predictions for more than 84% of the patients tested. Importantly, our method provides accurate predictions (>0.86 precision in cross validation) for major disease categories, including infectious and parasitic diseases, endocrine and metabolic diseases and diseases of the circulatory systems. Our performance applies to both chronic and acute diagnoses.

**Conclusions:**

Our results suggest that one can harness the wealth of population-based information embedded in electronic health records for patient-specific predictive tasks.

## Background

Over several decades, the vision of automatic systems assisting and supporting clinical decisions produced a plethora of clinical decision support systems [1-4], including diagnostic decision support systems for inferring patient diagnosis. These methods typically focus on a single patient and apply manually or automatically constructed decision rules to produce a diagnosis [2,5,6]. At the same time, health care is undergoing tremendous changes as medical information is digitized and archived in a structured fashion. Electronic health records (EHRs) promise to revolutionize the processes by which patients are administered, hospitalized and discharged [7], improve safety [8] and allow the conduct of post-hospitalization outcome research [9]. This large corpus of population-based records is increasingly used in the context of clinical decision making for the individual patient [10]. Nevertheless, there still seems to be no consistent association between EHRs and clinical decision support systems (CDSS) and better quality of care [11].

Recently, several methods have been released for predicting certain patient outcomes using large cohorts of patients. Two such examples are the detection of heart failure more than six months before the actual date of clinical diagnosis [12] and inference of patient prognosis based on patient similarities [13]. These methods, however, use the patient diagnosis for the learning task.

In this paper, we address a different, fundamental challenge – can we leverage the corpus of EHR patient data, even with well-documented quality issues [14], to infer the discharge diagnosis of patients using minimal medical data upon hospitalization. We introduce an automated method that exploits patient records for inferring an individual patient discharge diagnosis. For this task, we use basic patient-specific information gathered at admission, including medical history, blood tests, electrocardiography (ECG) results and demographics to identify similar patients, subsequently predicting patient outcomes. We test our method on two diverse sets of patients admitted to internal medicine departments in large medical centers in the United States and Israel, obtaining high precision and recall, suggesting that such systems may eventually be useful in the setting of assisting physicians with medical decisions, hospital planning and short-term resource allocation.

## Methods

### Data description

We obtained two EHR datasets from two hospitals: (i) 9,974 patients with 15,498 admissions, admitted in several wards belonging to internal medicine (for example, cardiology, oncology) or neurology over the course of two years from the Stanford Medical Center, CA, USA (USA dataset); and (ii) 5,513 patients with 7,070 admissions in internal medicine wards at the Rabin Medical Center, Israel between May 2010 and February 2012 (660 days; ISR dataset). Each dataset includes patient demographics (gender and age), medical history (International Classification of Diseases, Clinical Modification codes (ICD-9-CM) from past in- and out-patient encounters) and hospitalization specific information including blood test results and discharge diagnoses, coded as ICD-9 codes. A subset of the patients in the USA dataset includes ECG measurements, while the ISR dataset (7,261 patients) also contains ICD codes assigned upon admission. The USA dataset includes 86 commonly administered blood tests (after filtering, see below) and the ISR dataset includes 19 blood tests. Both patient cohorts include only urgent (non-elective) admissions and a roughly equal number of females and males. Both datasets cover the entire adult age spectrum (USA patients range between 15 and 90 years and ISR patients between 20 and 110), but the ISR cohort is skewed towards older patients (USA median age is 63 and ISR is 73, where 82% of ISR patients are above 60 while only 55% of the USA patients are).

In addition, we obtained records of the Healthcare Cost and Utilization Project (HCUP) of the Nationwide Inpatient Sample (NIS) of 2009 which contains more than 55 million associations between 5.8 million patients and 1,125 third level discharge ICD codes. The latter data were used to enhance the computation of ICD similarities, as described below.

The ICD codes in the EHR data included 469 (USA) and 396 (ISR) third level ICD codes (diagnostic and procedural codes). We excluded supplementary classification codes (codes starting with E or V) and several first level categories including complications of pregnancy (630 to 679) and codes in the range 740 to 999 for being uninformative (for example, general symptoms), a known condition (for example, congenital anomalies) or incidental conditions (for example, injuries or poisoning). We retained supplementary classification codes V40 to V49 –‘persons with a condition influencing their health status’ for being indicative of procedures a patient underwent.

As a sanity check, we extracted the ICD codes that were enriched in patients with extreme blood test values relative to other patients (hypergeometric test, false discovery rate (FDR) = 0.01) and verified that these corresponded to common knowledge associations, for example, various ICDs coding for cancer are enriched within patients with high lactic dehydrogenase values [15] or the troponin-*t* test is indicative of acute myocardial infarction [16] [See Additional file 1: Table S1 for the full association list].

The patients were de-identified by using a randomly generated patient id. The study was approved by the Institutional Review Board of Stanford and by the Helsinki Committee of the Rabin Medical Center.

### Similarity measure construction

In order to infer patient diagnosis, we computed a set of ten patient similarities. We computed two ICD similarity measures (1–2) and eight similarity measures between hospitalizations (3–10). All similarity measures were normalized to the range [0, 1]. We used the following ICD code similarities:

(1) ICD code similarity: We used the levels of the ICD codes in the ICD coding hierarchy to measure the similarity between ICD codes c_i_ and c_j_ as $S\left(c_{i},c_{j}\right)=\frac{\mathrm{NCA}\;\left(c_{i},c_{j}\right)}{\#\mathrm{levels}}$ , where NCA is the level of the nearest common ancestor and #levels are the number of levels in the ICD hierarchy (five levels) (see [17] for similar measures). When using third level codes, the number of levels equals three (the third, fourth and fifth levels).

(2) Empirical co-occurrence frequency: We used the HCUP data to compute empirical co-occurrences between ICD codes. Computing the number of co-occurrences of an ICD pair across all patients, we first computed the Jaccard score [18] between each pair. In order to transform the Jaccard score to a similarity measure, we randomly shuffled the associations of ICD codes to patients, keeping the overall ICD distribution as well as the per-patient ICD counts fixed. We then computed the similarity as the percentage of times the co-occurrence score was higher than the random shuffles.

### We used the following inter-patient similarity measures

(3–4) Medical history: Each patient may possess medical history from three sources: (i) past encounters with local health providers (digitally connected to the medical center); (ii) discharge codes of past hospitalizations; and (iii) personal history ICD codes provided in the current hospitalization (ICD codes V01to V15, V40 to V49 and V87). The union of these three sources constitutes the patient medical history profile. To compute the similarity of two such profiles, we form a bipartite graph over the member ICD codes, connecting two codes in the two profiles by an edge whose weight is the similarity between the codes. Our similarity score is the value of a maximal matching in this graph normalized by the smaller history set size. We performed the maximal matching computation using either of the two ICD similarity measures, resulting in two similarity measures.

(5–6) Blood test similarity: We used only the chronologically first blood test of each type, performed upon admission for each hospitalization, retaining only blood test results obtained during the first three days of hospitalization. We filtered blood tests that were performed in less than 5% of the hospitalizations and those for which the difference in distribution between patients with the same diagnosis and patients without shared diagnosis was not statistically significant (Wilcoxon ranked sum test, FDR <0.01). This left us with 86 blood tests for the USA dataset and 19 blood tests for the ISR set. Each blood test was then normalized by converting it to a *z*-score, mean and standard deviation measured across the initial blood tests of all patients. Most of the patients had undergone only a partial set of the tests. We removed patients having fewer than three available blood tests and computed the similarity between a pair of hospitalizations based on the values of the blood tests common to the two hospitalizations, where patients sharing fewer than three blood tests between them received the minimal similarity score of zero. We formed two types of similarities: (i) using the entire set of common blood test array between any two hospitalizations, we computed the Euclidean distance between the *z*-score vectors, normalized by their length; and (ii) the average of differences in absolute values between the blood tests with the highest z-score for each patient. The distance D_ij_ between patients i and j was converted to a similarity value by linear transformation.

(7–8) ECG similarity: The ECG values included eight interval values as well as the heart rate. Similarly to the blood tests, we used only the chronologically first measurement, performed upon admission for each hospitalization, obtained during the first three days of hospitalization. Each ECG measurement had undergone the same normalization and similarity construction as the blood tests.

(9) Age similarity: In order to give precedence to age differences in younger age, we computed the similarity between two patients *p*_*i*_ and *p*_*j*_ as

$$S\;\left(p_{i},p_{j}\right)=1-\frac{\left|p_{i}-p_{j}\right|}{\max\;\left(p_{i},p_{j}\right)}$$

(10) Gender similarity: defined as 1 if the two patients have the same gender and 0 otherwise.

### Combining similarity measures to classification features

The framework we used scores a hypothetical association according to its maximal similarity to a known, gold-standard, set of associations. In our case, we scored associations between hospitalization records and ICD codes based on the highest similarity to the known discharge codes in the background corpus of previously hospitalized patients (disregarding similarities to previous hospitalizations of the same patient). Specifically, the features used to classify hospitalization-primary discharge ICD code pairs were constructed from scores computed for each combination of an ICD-similarity measure and a similarity measure between patient hospitalizations (see previous section for details), resulting in 16 features overall (12 without the ECG similarities). For each such pair of similarity measures, the score of a potential discharge code *I* for a given hospitalization *H* is computed by considering the similarity to known discharge codes associated with other hospitalizations (excluding other hospitalizations of the same patient) (*I’ and H’)*. The computation is done as follows: First, for each known associations *(H’,I’)* we compute the inter-hospitalization similarity *S(H,H’)* and the ICD codes similarity *S(I,I’)*. Next, we follow the method of [19] to combine the two similarities to a single score by computing their geometric mean. Thus:

(1) $$\mathrm{Score}\left(H,I\right)=\max_{H',I'\neq H,I}\sqrt{S\left(H,H'\right)\cdot S\left(I,I'\right)}$$

### Performance evaluation

We used the MATLAB implementation of the logistic regression classifier (glmfit function with binomial distribution and logit linkage) for the prediction task. We used a 10-fold cross validation scheme to evaluate the precision of our prediction algorithm. The training set used for the cross validation included 41,036 USA associations between hospitalizations and discharge codes and 14,506 ISR associations. We considered two types of negative sets, the same size as the positive set in each training set: (i) randomly sampling for each patient a diagnosis from the 469 (USA) or 396 (ISR) third level ICD codes (excluding true diagnoses for that patient), termed ‘pre-admission’; and (ii) randomly sampling a set of potential release codes for each hospitalization, termed ‘post-admission.’ Specifically for the second negative set scenario, we inspected the available admission diagnoses reported upon hospitalization (lacking from the USA dataset) and included the set of discharge diagnoses of all the patients who shared the same admission diagnosis (excluding the true discharge diagnosis for that hospitalization). As an example, the potential negative set for a patient admitted with chest pain includes the discharge diagnoses of all other patients admitted with chest pain, excluding the true final diagnoses of that patient. Additionally, we removed self-similarities of patients (that is, similarities between hospitalizations of the same patient) to avoid bias for patients with recurrent admissions. To obtain robust area under the curve (AUC) score estimates, we performed 10 independent cross validation runs, selecting a different negative set and a different random partition of the training set to 10 parts in each; we then averaged the resulting AUC scores. Expectedly, taking a negative set of size five, ten or twenty times the size of the positive set had a negligible effect on the resulting AUC score (AUC difference less than 0.002).

In order to apply our method in a scenario that mimics the admission of new patients, we split the hospitalizations into training and validation subsets. For the ISR data, we used the available admission date to select hospitalizations that spanned the first year of our data (July 2010 to June 2011) as our training set and validated on hospitalizations occurring in the subsequent 211 days, totaling 999 hospitalizations. For the USA data, we split the data into train and test sets (two thirds and a third, respectively) using the available sequential ordering of their admission dates. As with the cross-validation scheme, we masked similarities between hospitalizations of the same patient. We computed the precision of our predictions by counting the number of patients for which the top predicted discharge code was the same as one of its true diagnoses. Similarly, we also computed the performance when testing whether the true discharge code of a patient appeared in the top two predictions, top three and up to the top ten predictions per patient.

In order to identify ICD codes that are significantly correctly predicted, we compared the number of correct predictions for each ICD code against a background of 10^5^ randomly shuffled patient-diagnosis associations sets.

## Results

### The inference framework

**Figure 1:**
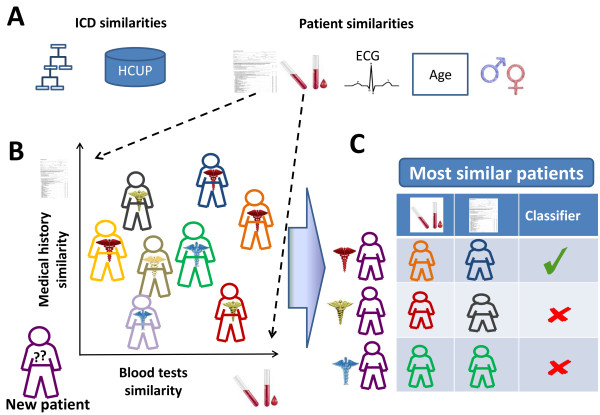
**A schematic view of the method.** Similarities between ICD codes and between hospitalizations are computed **(A)**. A new patient is scored according to the most similar patients with a certain diagnosis **(B)**. A classifier is applied to select the top scoring diagnoses for this patient **(C)**. ICD, International Classification of Diseases.

Our objective was to test whether a minimal amount of patient information, available upon admission in EHRs, can be integrated with a background corpus of previous patients to infer the patient’s primary discharge ICD codes (including both diagnoses and procedure codes). The patient information we used for this task includes medical history, the results of the first administered blood and ECG tests and demographics (Methods). To this end, we defined novel diagnosis- and patient-similarity measures, allowing us to exploit the similarity-based inference framework of [19] for inferring associations between hospitalization records and primary discharge ICD codes (see Methods and Figure 1 for an overview).

In order to gain insights about the global properties of the medical history, blood test and ECG similarities, we first examined the networks formed by associating an individual patient with the closest matching patient in the historical database. Interestingly, the networks formed by these similarities show marked differences (consistent across the two EHR datasets). While medical history similarities tend to connect patients into big clusters, blood test and ECG similarities display highly disconnected sub-networks [See Additional file 2: Figures S1A-C and Additional file 3: Figure S2A-B]. The integration of similarity measures with markedly different properties boosts classification performance (as displayed in Additional file 3: Figure S2).

### Prediction of discharge ICD-9 codes

We focused on inferring the primary discharge codes for the hospitalization, as they encompass the most crucial piece of information for the caring physician. Due to information content and ICD code usage differences between the two datasets, we train and predict on each dataset independently (see also Discussion for expansion). Our EHR datasets included a set of ranked discharge codes assigned by hospital specialists based on coded and unstructured clinical data in the patient record. We selected a gold standard of ‘primary’ discharge codes consisting of the two top-ranked discharge codes per patient. In the case of the ISR dataset we added a sparse set of release codes assigned by the physician (accompanying the free-text release notes) totaling 2.2 ± 1.2 codes per patient on average. Overall, our set included 469 and 396 third level ICD diagnostic and procedural codes for the USA and ISR datasets, respectively (Methods).

In order to validate our predictions, we first applied a 10-fold cross validation scheme. In selecting the negative set, we considered two scenarios: (i) sampling of the entire set of false ICD codes (termed ‘pre-admission’, see Methods); and (ii) a more realistic case, available only in the ISR dataset, in which we sample only from the potential discharge diagnoses that a physician might consider based on the patient admission diagnoses (termed ‘post-admission’). We summarize the cross-validation results for several scenarios in Table 1, showing that our results are highly robust to differences in information content and across datasets (AUC >0.88). However, the ‘post-admission’ scenario proved to be a more demanding task due to the need to differentiate between more similar diagnoses, obtaining a lower AUC score (AUC = 0.77). More importantly, the highest ranking prediction for each hospitalization was correct in 93% (± 0.4%) and 92% (± 0.3%) for the USA and ISR datasets, respectively (85% ± 0.4% in the post-admission scenario).

**Table 1:** Performance in cross-validation experiments

**Cross validation scenario**	**AUC**	**Best F1 measure**	**AUC, non-chronic patients**
USA, 10K patients with ECG data	0.9 ± 9E^-4^	0.83 ± 0.001	0.89 ± 0.0009
USA, 15K patients without ECG data	0.89 ± 7E^-4^	0.82 ± 9E^-4^	0.88 ± 0.001
ISR, pre-admission scenario	0.88 ± 0.001	0.81 ± 0.001	0.86 ± 0.002
ISR, post-admission scenario	0.77 ± 0.002	0.73 ± 0.002	0.76 ± 0.003
Merged datasets	0.87± 9E^-^^4^	0.81 ± 7E^-4^	0.86 ± 0.002

*AUC*, area under the curve; *ECG*, electrocardiography; *ISR*, Israel; *USA*, United States.

**Figure 2:**
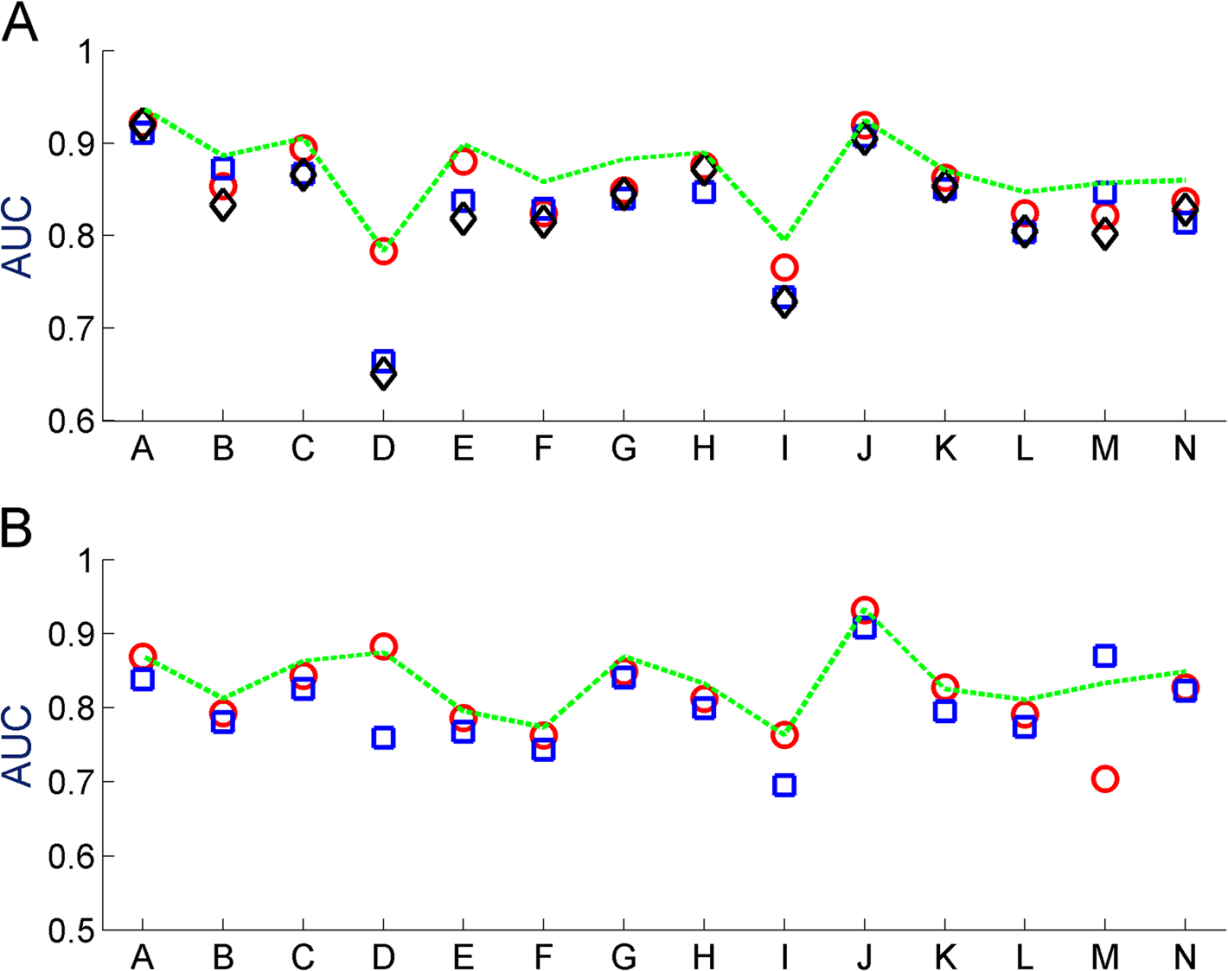
**AUC scores for ICD level 1 categories.** AUC scores using only the blood test features (red circles), medical history (blue squares), ECG measurements (black diamonds) and all features (dashed green line) are displayed for the USA **(A)** and ISR **(B)** datasets across ICD level 1 categories: Infectious And Parasitic Diseases **(A)**, Neoplasms **(B)**, Endocrine, Nutritional And Metabolic Diseases, And Immunity Disorders **(C)**, Diseases Of The Blood And Blood-Forming Organs **(D)**, Mental Disorders **(E)**, Diseases Of The Nervous System And Sense Organs **(F)**, Diseases Of The Circulatory System **(G)**, Diseases Of The Respiratory System **(H)**, Diseases Of The Digestive System **(I)**, Diseases Of The Genitourinary System **(J)**, Diseases Of The Skin And Subcutaneous Tissue **(K)**, Diseases Of The Musculoskeletal System And Connective Tissue **(L)**, Supplementary Classification Of Factors Influencing Health Status And Contact With Health Services **(M)** and Classification Of Procedures **(N)**. AUC, area under the curve; ECG, electrocardiography; ICD, International Classification of Diseases.

Analyzing the contribution of each feature, we observe that the features involving the hierarchy-based ICD similarity outperformed features built with empirical co-occurrence ICD similarity. Analyzing the classification power of each of the inter-patient similarity measures, we found that none was sufficient for obtaining the overall AUC, with blood tests achieving slightly higher results than medical history or ECG as standalones (AUC <0.85, Additional file 3: Figure S2). It is noteworthy that the medical history feature built using the empirical ICD similarity performed much better in the USA dataset than the ISR dataset, possibly owing to the fact that the empirical ICD similarities were built using an (independent) USA-based patient cohort. We further computed the AUC scores per feature (blood tests, medical history or ECG measurements) across different first level ICD categories (Figure 2). Blood tests perform significantly better than medical history and ECG as classifiers in most of the categories (Wilcoxon ranked sum test, corrected for multiple hypotheses with FDR <0.01), with a notable performance increase in diseases of the blood and of the digestive system. Interestingly, we find that blood tests perform better in mental disorders than medical history. Indeed, the majority of the patients discharged with mental disorders in our cohorts had no mention of mental disorder in their medical history (69% and 82% in the USA and ISR datasets, respectively). Medical history performed better for neoplasms in the USA dataset, while ECG had equivalent performance to blood tests for infectious and parasitic diseases and diseases of the respiratory systems.

To ensure that our method is not limited to detecting only chronic patients, which we defined as ones for whom the discharge diagnosis appears also in their medical history (including previous hospitalizations), we verified that we achieve a similar performance when applying our method to a set of 9,990 USA or 5,838 ISR hospitalizations which include only non-chronic cases (Table 1). Expectedly, blood tests perform significantly better than medical history in this set for all first level ICD categories (FDR <0.01).

### Prospective validation

**Figure 3:**
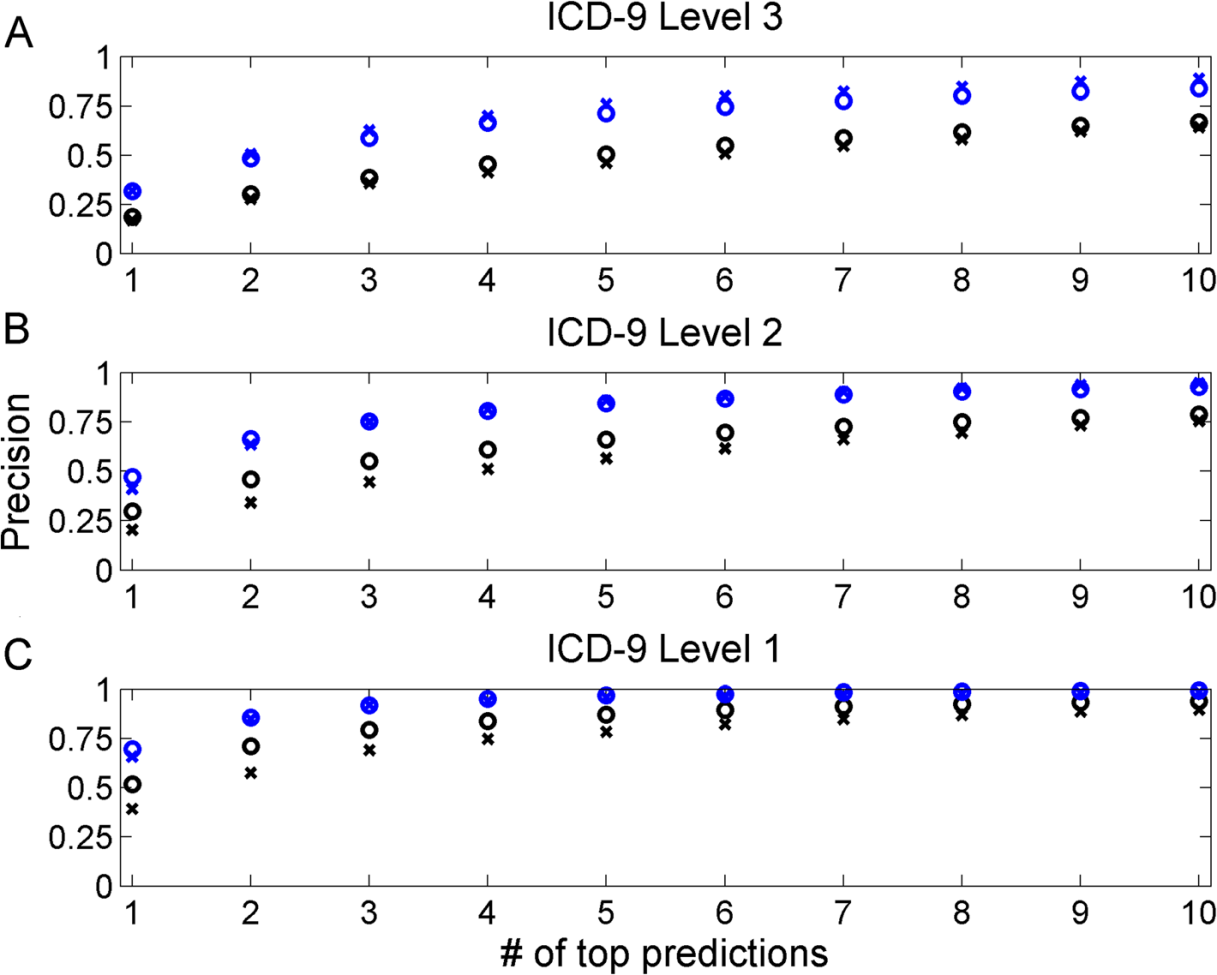
**Prediction precision for recent hospitalizations.** The prediction precision for primary discharge codes (black) and all discharge codes (blue) for the USA data (circles) and the ISR data (crosses) as a function of the number of top ranked predictions per patient. Precision is measured for ICD level 1 **(A)**, level 2 **(B)** and level 3 **(C)**. ICD, International Classification of Diseases; ISR, Israel; USA, United States.

Next, we applied our method in a scenario that mimics the admission of new patients. We split the hospitalizations into training and validation subsets, based on admission date when available (Methods). In the following, we report first the USA dataset performance and the ISR performance is provided in parentheses for clarity. As we focus on predicting at least one primary diagnosis per patient, we measure our performance by computing the percentage of patients with at least one correct prediction (that is, precision). While the top predicted discharge code was correct for 18% (17%) of the patients, the top ten predictions contained a correct discharge code for 67% (64%) of the patients. We note that the task here is more challenging than the previous ‘cross-validation’ one since the latter evaluates a specific set of options for ICD codes (those in the test set) while here we evaluate all possible codes as we have no prior information for a new patient. One reason for the lower precision lies in the fact that discharge diagnosis codes include also ‘secondary’ discharge codes, ranked lower than the top two discharge codes for a patient. Since the distinction between primary (top discharge codes and physician release codes) and secondary (additional discharge codes) is done manually and is subjective, we also checked the prediction precision relative to the complete set of discharge codes, including both primary codes and secondary codes (the latter not appearing in the training set) to find that our top prediction was correct for 32% of the patients (both datasets) with 84% (89%) of patients with at least one correct hit within the top ten predictions (Figure 3A). For example, we predicted diabetes mellitus for a patient who indeed had that condition; however it was not marked as the primary diagnosis. For comparison, we tested the precision against 1,000 sets of randomly shuffled associations between diagnoses and patients (maintaining the distribution of the ICDs and the number of diagnoses per patient), verifying that none of the shuffled associations obtained comparable precision (*P* <0.001).

As a physician can likely also benefit from a more coarse classification, we checked the precision in predicting the second and first level of the ICD (Figures 3B and 3C, respectively). The top prediction was accurate for 47% (41%) of the patients when considering second level ICD codes and 70% (66%) when considering the first level codes (including also non-primary codes). Similarly, 93% (95%) of the patients had the correct second level ICD code in their top ten predictions (and 99% (98%) the correct first level code). Manually examining the hospitalizations for which we failed to predict the correct second level of the ICD (spanning 7% (5%) of the patients), we found that several of our predictions, while not an exact match, had a known association to the correct diagnoses. For example, a patient with acute bronchitis was predicted to have chronic bronchitis (noting that this patient had no such chronic condition mentioned in his medical history). Other examples include prediction of heart failure for two (ISR) patients (a man and a woman) diagnosed with acute myocardial infarction, and the latter often supersedes the former [20] and prediction of episodic mood disorders for a (USA) patient with depressive disorder.

Finally, we analyzed the prediction performance over the different diagnoses. Expectedly, we found a high correlation (Pearson correlation, rho = 0.9, *P* <2e^-164^ (0.85, *P* <e^-131^)) between the number of patients in the training set with a certain ICD code and the success rate in predicting it among the top ten predictions [See Additional file 4: Figure S3]. We identified 33 (17) ICD codes that were significantly correctly predicted in each EHR dataset (FDR <0.05, Methods and Additional file 5: Table S1). Six ICD diagnosis codes were common to both datasets: diabetes mellitus, pneumonia, bronchitis, diseases of white blood cells, kidney failure and disorders of urethra and urinary tract, while an additional nine (six USA and three ISR codes) were under the same second level ICD (metabolic disorders, diseases of the blood, hypertensive disease and chronic bronchitis). Additionally, some enriched ICDs belonged to similar categories, such as heart related conditions (for example, cardiomyopathy, cardiac dysrhythmias and heart failure in the USA dataset versus chronic ischemic heart disease in the ISR dataset). In contrast, ICD codes that had no successful prediction even when allowing for first level of the ICD match generally suffered from low representation in the training data [see Additional file 3: Figure S3] and were typically accompanied by diagnoses with higher success rates. One such example is gastrointestinal hemorrhage, appearing in nine patients in our validation set (ISR dataset). This diagnosis was accompanied by other diagnoses in all these cases and, indeed, for seven of these patients we managed to predict all their additional diagnoses. Figures 2 and Additional file 6: Figure S4 display the AUC scores and prediction precision across different first level ICD categories for the cross and prospective validations, respectively.

## Discussion

We used patient cohorts from two different hospitals. However, we trained and provided predictions for each dataset independently. This was done for three reasons: (i) combining the two datasets ignores information available in only one dataset (for example ECG data or blood tests that appear in only one set); (ii) the ICD codes, primarily used for billing purposes, are often biased due to the health system used in each country; and (iii) different sources of medical history (that is, outpatient versus inpatient facilities) display lower agreement between patients from different health systems. Indeed, we observed that merging the two datasets degraded the performance to that of the worse performing dataset (ISR, see Table 1).

In order to assess the potential benefits to a clinician, we looked at predictions that could be considered surprising with regard to the admission diagnoses (available in the ISR dataset). We found multiple examples in which the admission diagnosis contained only general symptoms and our method correctly predicted the true discharge diagnosis. We describe here two such examples: (i) a female patient who was admitted with an unspecified anemia (ICD code 285.9) was correctly predicted for cardiac dysrhythmias (427). Irregular heartbeat is one of the many symptoms of anemia but not a predictive one [21]; and (ii) a female patient was admitted with fever (780.6) and was correctly predicted for acute myocardial infarction (410). Notably, fever is not a common symptom for acute myocardial infarction [22].

Finally, analyzing our performance, we note that while our method provided high quality predictions in cross validation, it is likely to display lower performance in predicting conditions that evolve substantially over time and conditions that are rare in the population. We observe that high level ICD categories that achieve relative high precision are typically abundant in our data (above 6% (USA) and 4% (ISR) of the patients), including diseases related to endocrine, circulatory, respiratory and genitourinary systems (Figure 3). In contrast, lower precision is obtained for high level ICD categories which generally have a low representation in our data and are typically complex (for example, neoplasms). A larger and richer EHR data could enhance our prediction precision in these cases also. Specifically, a very large corpus of patients might introduce more of the currently rare cases and having a larger temporal range within the corpus would allow for richer representations of the medical history. This assumption is strengthened by the fact that the USA dataset is obtained from a tertiary care facility and, thus, harbors more ‘hard’ cases. Yet this dataset obtained better performance due to a larger corpus of patients and more information on each patient than the ISR dataset which is from a primary and secondary care facility. One reason may be that only a small subset of the blood tests was available for each patient in the ISR dataset, limiting the computation of similarity between patients and the ability to account for rarer test types. A fuller set of tests allows the computation of more accurate patient similarities.

## Conclusions

Our results demonstrate that a large corpus of patient data can be exploited to predict the likely discharge diagnoses for a new patient. We introduced a general method for performing such an inference using information from past hospitalizations. Our method computes patient similarity measures and requires a minimal set of such measures, including medical history, blood tests performed upon admission and demographics. It is readily extensible to use the results of other admission information, such as ECG tests, as shown for the USA dataset and potentially, in the future, medical images and patient genomic information (for example, gene expression measurements or single nucleotide polymorphism data).

Our method is a stepping stone for the full exploitation of large population-based data sets. We recognize that the introduction of new decision support modalities requires careful analysis of physician and health-care system workflows and introduction of the information at the most pertinent decision points. However, it is clear that the emerging infrastructure of electronic patient information will provide not only better information about quality of care and guidance for policy but will be able to improve the care of the individual, benefitting from the aggregated information of previous patients.

## Abbreviations

AUC: Area under the curve; ECG: Electrocardiography; EHR: Electronic health records; FDR: False discovery rate; HCUP: Healthcare Cost and Utilization Project; ICD: International Classification of Diseases.

## Competing interests

The authors declare that they have no competing interests.

## Authors’ contributions

AG and RS conceived the paper; AG performed the analysis and wrote the draft; GS obtained the data, and aided in pre-processing; GS, ER, RA and RS participated in the writing of the paper. All authors read and approved the final manuscript.

## Pre-publication history

The pre-publication history for this paper can be accessed here:

http://www.biomedcentral.com/1741-7015/11/194/prepub

## Supplementary Material

Additional file 1: Table S2 ICD codes enriched in extreme valued blood tests. Click here for file

Additional file 2: Figure S1 Networks of patient similarities. The similarity between patients based on medical history (A), blood test (B) and ECG (C) data. Click here for file

Additional file 3: Figure S2 The performance of individual features in cross validation. Displayed are individual feature AUC scores for the USA data (Red) and ISR data (blue). The abbreviated feature combinations include: ICD hierarchy-based similarity (I1), ICD empirical similarity (I2), Age (A), Gender (G), blood tests- average difference (BT1), blood tests-difference between extremes (BT2), ECG tests- average difference (ECG1), ECG tests-difference between extremes (ECG2), medical history (MH1) and medical history – empirical ICD similarity based (MH2). Click here for file

Additional file 4: Figure S3 The precision in predicting ICD codes as a function of the number of patients in the training set for the USA (A) and ISR (B) datasets. Click here for file

Additional file 5: Table S1 Easy to predict ICD codes. All p-values are FDR corrected. Click here for file

Additional file 6: Figure S4 Prediction precision for ICD level 1 categories. Precision values (blue) and relative prevalence (red) are displayed for the USA (A) and ISR (B) datasets across ICD level 1 categories: Infectious And Parasitic Diseases (A), Neoplasms (B), Endocrine, Nutritional And Metabolic Diseases, And Immunity Disorders (C), Diseases Of The Blood And Blood-Forming Organs (D), Mental Disorders (E), Diseases Of The Nervous System And Sense Organs (F), Diseases Of The Circulatory System (G), Diseases Of The Respiratory System (H), Diseases Of The Digestive System (I), Diseases Of The Genitourinary System (J), Diseases Of The Skin And Subcutaneous Tissue (K), Diseases Of The Musculoskeletal System And Connective Tissue (L), Supplementary Classification Of Factors Influencing Health Status And Contact With Health Services (M) and Classification Of Procedures (N). Click here for file
