# Supplementary material for: A method for inferring medical diagnoses from patient similarities
Source: BMC Med. 2013 Sep 2;11:194. doi: 10.1186/1741-7015-11-194 (PMC3844462; doi:10.1186/1741-7015-11-194)
Supplement: Additional file 1: Table S2 — ICD codes enriched in extreme valued blood tests. [file 1741-7015-11-194-S1.pdf]

USA dataset

Blood test	ICD code	ICD description
PLATELET COUNT	038	Septicemia
PLATELET COUNT	197	Secondary malignant neoplasm of respiratory and digestive systems
PLATELET COUNT	511	Pleurisy
PLATELET COUNT	567	Peritonitis and retroperitoneal infections
PLATELET COUNT	707	Chronic ulcer of skin
PLATELET COUNT	Z54	Other operations on abdominal region
PLATELET COUNT	Z86	Operations on skin and subcutaneous tissue
PLATELET COUNT	Z99	Other nonoperative procedures
HEMATOCRIT	204	Lymphoid leukemia
HEMATOCRIT	205	Myeloid leukemia
HEMATOCRIT	280	Iron deficiency anemias
HEMATOCRIT	282	Hereditary hemolytic anemias
HEMATOCRIT	283	Acquired hemolytic anemias
HEMATOCRIT	284	Aplastic anemia and other bone marrow failure syndromes
HEMATOCRIT	285	Other and unspecified anemias
HEMATOCRIT	288	Diseases of white blood cells
HEMATOCRIT	455	Hemorrhoids
HEMATOCRIT	531	Gastric ulcer
HEMATOCRIT	532	Duodenal ulcer
HEMATOCRIT	537	Other disorders of stomach and duodenum
HEMATOCRIT	569	Other disorders of intestine
HEMATOCRIT	578	Gastrointestinal hemorrhage
HEMATOCRIT	Z38	Incision, excision, and occlusion of vessels
HEMATOCRIT	Z41	Operations on bone marrow and spleen
HEMATOCRIT	Z44	Other operations on stomach
HEMATOCRIT	Z45	Incision, excision, and anastomosis of intestine
HEMATOCRIT	Z99	Other nonoperative procedures
HEMOGLOBIN	205	Myeloid leukemia
HEMOGLOBIN	280	Iron deficiency anemias
HEMOGLOBIN	283	Acquired hemolytic anemias
HEMOGLOBIN	284	Aplastic anemia and other bone marrow failure syndromes
HEMOGLOBIN	285	Other and unspecified anemias
HEMOGLOBIN	531	Gastric ulcer
HEMOGLOBIN	532	Duodenal ulcer
HEMOGLOBIN	537	Other disorders of stomach and duodenum
HEMOGLOBIN	569	Other disorders of intestine
HEMOGLOBIN	578	Gastrointestinal hemorrhage
HEMOGLOBIN	Z41	Operations on bone marrow and spleen
HEMOGLOBIN	Z44	Other operations on stomach
HEMOGLOBIN	Z45	Incision, excision, and anastomosis of intestine
HEMOGLOBIN	Z99	Other nonoperative procedures

RBC	205	Myeloid leukemia
RBC	280	Iron deficiency anemias
RBC	282	Hereditary hemolytic anemias
RBC	283	Acquired hemolytic anemias
RBC	284	Aplastic anemia and other bone marrow failure syndromes
RBC	285	Other and unspecified anemias
RBC	288	Diseases of white blood cells
RBC	531	Gastric ulcer
RBC	532	Duodenal ulcer
RBC	537	Other disorders of stomach and duodenum
RBC	578	Gastrointestinal hemorrhage
RBC	Z38	Incision, excision, and occlusion of vessels
RBC	Z41	Operations on bone marrow and spleen
RBC	Z44	Other operations on stomach
RBC	Z45	Incision, excision, and anastomosis of intestine
RBC	Z99	Other nonoperative procedures
WBC	038	Septicemia
WBC	204	Lymphoid leukemia
WBC	205	Myeloid leukemia
WBC	518	Other diseases of lung
WBC	Z41	Operations on bone marrow and spleen
WBC	Z99	Other nonoperative procedures
MCV	070	Viral hepatitis
MCV	280	Iron deficiency anemias
MCV	517	Lung involvement in conditions classified elsewhere
MCV	571	Chronic liver disease and cirrhosis
MCV	572	Liver abscess and sequelae of chronic liver disease
MCV	Z45	Incision, excision, and anastomosis of intestine
MCV	Z54	Other operations on abdominal region
RDW	070	Viral hepatitis
RDW	197	Secondary malignant neoplasm of respiratory and digestive systems
RDW	280	Iron deficiency anemias
RDW	282	Hereditary hemolytic anemias
RDW	284	Aplastic anemia and other bone marrow failure syndromes
RDW	511	Pleurisy
RDW	517	Lung involvement in conditions classified elsewhere
RDW	518	Other diseases of lung
RDW	572	Liver abscess and sequelae of chronic liver disease
RDW	Z39	Other operations on vessels
RDW	Z41	Operations on bone marrow and spleen
RDW	Z45	Incision, excision, and anastomosis of intestine
RDW	Z54	Other operations on abdominal region
RDW	Z99	Other nonoperative procedures

NEUT, ABS	038	Septicemia
NEUT, ABS	486	Pneumonia, organism unspecified
NEUT, ABS	507	Pneumonitis due to solids and liquids
NEUT, ABS	518	Other diseases of lung
NEUT, ABS	Z38	Incision, excision, and occlusion of vessels
NEUT, ABS	Z96	Nonoperative intubation and irrigation
LYM, ABS	204	Lymphoid leukemia
MONO, ABS	038	Septicemia
MONO, ABS	282	Hereditary hemolytic anemias
MONO, ABS	486	Pneumonia, organism unspecified
EOS, ABS	282	Hereditary hemolytic anemias
EOS, ABS	585	Chronic kidney disease (CKD)
EOS, ABS	Z39	Other operations on vessels
BASOS, ABS	282	Hereditary hemolytic anemias
NEUT, %	204	Lymphoid leukemia
NEUT, %	205	Myeloid leukemia
NEUT, %	284	Aplastic anemia and other bone marrow failure syndromes
NEUT, %	288	Diseases of white blood cells
NEUT, %	291	Alcohol-induced mental disorders
NEUT, %	296	Episodic mood disorders
NEUT, %	303	Alcohol dependence syndrome
NEUT, %	307	Special symptoms or syndromes, not elsewhere classified
LYM, %	204	Lymphoid leukemia
LYM, %	205	Myeloid leukemia
LYM, %	284	Aplastic anemia and other bone marrow failure syndromes
LYM, %	287	Purpura and other hemorrhagic conditions
LYM, %	288	Diseases of white blood cells
LYM, %	291	Alcohol-induced mental disorders
LYM, %	296	Episodic mood disorders
LYM, %	307	Special symptoms or syndromes, not elsewhere classified
MONO, %	070	Viral hepatitis
MONO, %	288	Diseases of white blood cells
MONO, %	572	Liver abscess and sequelae of chronic liver disease
MONO, %	Z54	Other operations on abdominal region
MONO, %	Z99	Other nonoperative procedures
TROPONIN I, POCT	410	Acute myocardial infarction
TROPONIN I, POCT	Z00	Procedures and interventions, Not Elsewhere Classified
TROPONIN I, POCT	Z36	Operations on vessels of heart
LACTATE, ISTAT	038	Septicemia
LACTATE, ISTAT	518	Other diseases of lung
LACTATE, ISTAT	Z96	Nonoperative intubation and irrigation

ISR dataset

Blood test	ICD code	ICD description
Standard Base Excess	584	Acute kidney failure
Standard Base Excess	585	Chronic kidney disease (CKD)
Bilirubin Total	070	Viral hepatitis
Bilirubin Total	157	Malignant neoplasm of pancreas
Bilirubin Total	571	Chronic liver disease and cirrhosis
Bilirubin Total	572	Liver abscess and sequelae of chronic liver disease
Bilirubin Total	576	Other disorders of biliary tract
Bilirubin Total	Z54	Other operations on abdominal region
Calcium	038	Septicemia
Calcium	197	Secondary malignant neoplasm of respiratory and digestive systems
Calcium	275	Disorders of mineral metabolism
Creatinine	403	Hypertensive chronic kidney disease
Creatinine	584	Acute kidney failure
Creatinine	585	Chronic kidney disease (CKD)
Creatinine	V45	Other postprocedural states
Creatinine	Z39	Other operations on vessels
Glucose	250	Diabetes mellitus
Glucose	270	Disorders of amino-acid transport and metabolism
Glucose	357	Inflammatory and toxic neuropathy
Glutamic Oxaloacetic Tran	573	Other disorders of liver
Glutamic Pyruvic Transam	573	Other disorders of liver
Glutamic Pyruvic Transam	576	Other disorders of biliary tract
Hco3	584	Acute kidney failure
Hemoglobin	238	Neoplasm of uncertain behavior of other and unspecified sites and tissues
Hemoglobin	280	Iron deficiency anemias
Hemoglobin	281	Other deficiency anemias
Hemoglobin	285	Other and unspecified anemias
Hemoglobin	535	Gastritis and duodenitis
Hemoglobin	553	Other hernia of abdominal cavity without mention of obstruction or gangrene
Hemoglobin	578	Gastrointestinal hemorrhage
Hemoglobin	Z99	Other nonoperative procedures
Lactic Dehydrogenase	172	Malignant melanoma of skin
Lactic Dehydrogenase	185	Malignant neoplasm of prostate
Lactic Dehydrogenase	197	Secondary malignant neoplasm of respiratory and digestive systems
Lactic Dehydrogenase	198	Secondary malignant neoplasm of other specified sites
Lactic Dehydrogenase	202	Other malignant neoplasms of lymphoid and histiocytic tissue
Lactic Dehydrogenase	204	Lymphoid leukemia
Lactic Dehydrogenase	208	Leukemia of unspecified cell type
Lactic Dehydrogenase	209	Neuroendocrine tumors
Lactic Dehydrogenase	410	Acute myocardial infarction

Absolute Neutrophil Coun	038	Septicemia
Absolute Neutrophil Coun	041	Bacterial infection in conditions classified elsewhere and of unspecified site
Absolute Neutrophil Coun	486	Pneumonia, organism unspecified
Absolute Neutrophil Coun	599	Other disorders of urethra and urinary tract
Phosphorus	038	Septicemia
Phosphorus	403	Hypertensive chronic kidney disease
Phosphorus	514	Pulmonary congestion and hypostasis
Phosphorus	584	Acute kidney failure
Phosphorus	585	Chronic kidney disease (CKD)
Phosphorus	586	Renal failure, unspecified
Phosphorus	Z96	Nonoperative intubation and irrigation
Platelet (PLT)	205	Myeloid leukemia
Platelet (PLT)	280	Iron deficiency anemias
Platelet (PLT)	287	Purpura and other hemorrhagic conditions
Platelet (PLT)	707	Chronic ulcer of skin
Po2	038	Septicemia
Po2	345	Epilepsy and recurrent seizures
Po2	733	Other disorders of bone and cartilage
Po2	Z79	Reduction of fracture and dislocation
Po2	Z81	Repair and plastic operations on joint structures
Po2	Z96	Nonoperative intubation and irrigation
Potassium	174	Malignant neoplasm of female breast
Potassium	276	Disorders of fluid, electrolyte, and acid-base balance
Potassium	403	Hypertensive chronic kidney disease
Potassium	584	Acute kidney failure
Potassium	586	Renal failure, unspecified
Sodium	253	Disorders of the pituitary gland and its hypothalamic control
Sodium	276	Disorders of fluid, electrolyte, and acid-base balance
Sodium	584	Acute kidney failure
Sodium	707	Chronic ulcer of skin
Troponin T	410	Acute myocardial infarction
White Blood Cells	038	Septicemia
White Blood Cells	204	Lymphoid leukemia
