# Supplementary figures and images for: A method for inferring medical diagnoses from patient similarities
Source: BMC Med. 2013 Sep 2;11:194. doi: 10.1186/1741-7015-11-194 (PMC3844462; doi:10.1186/1741-7015-11-194)

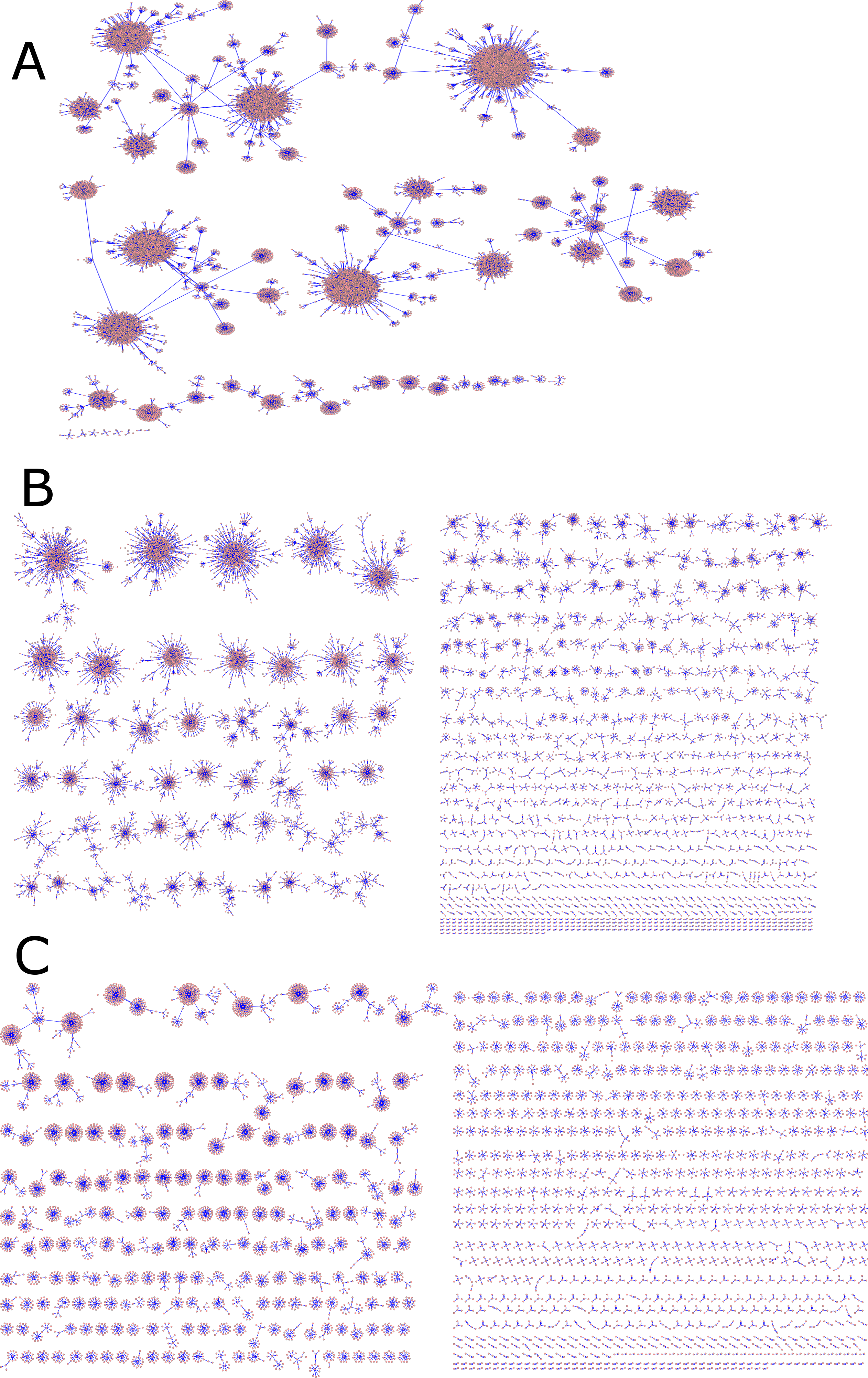

Supplement: Additional file 2: Figure S1 — Networks of patient similarities. The similarity between patients based on medical history (A), blood test (B) and ECG (C) data. [file 1741-7015-11-194-S2.tiff]

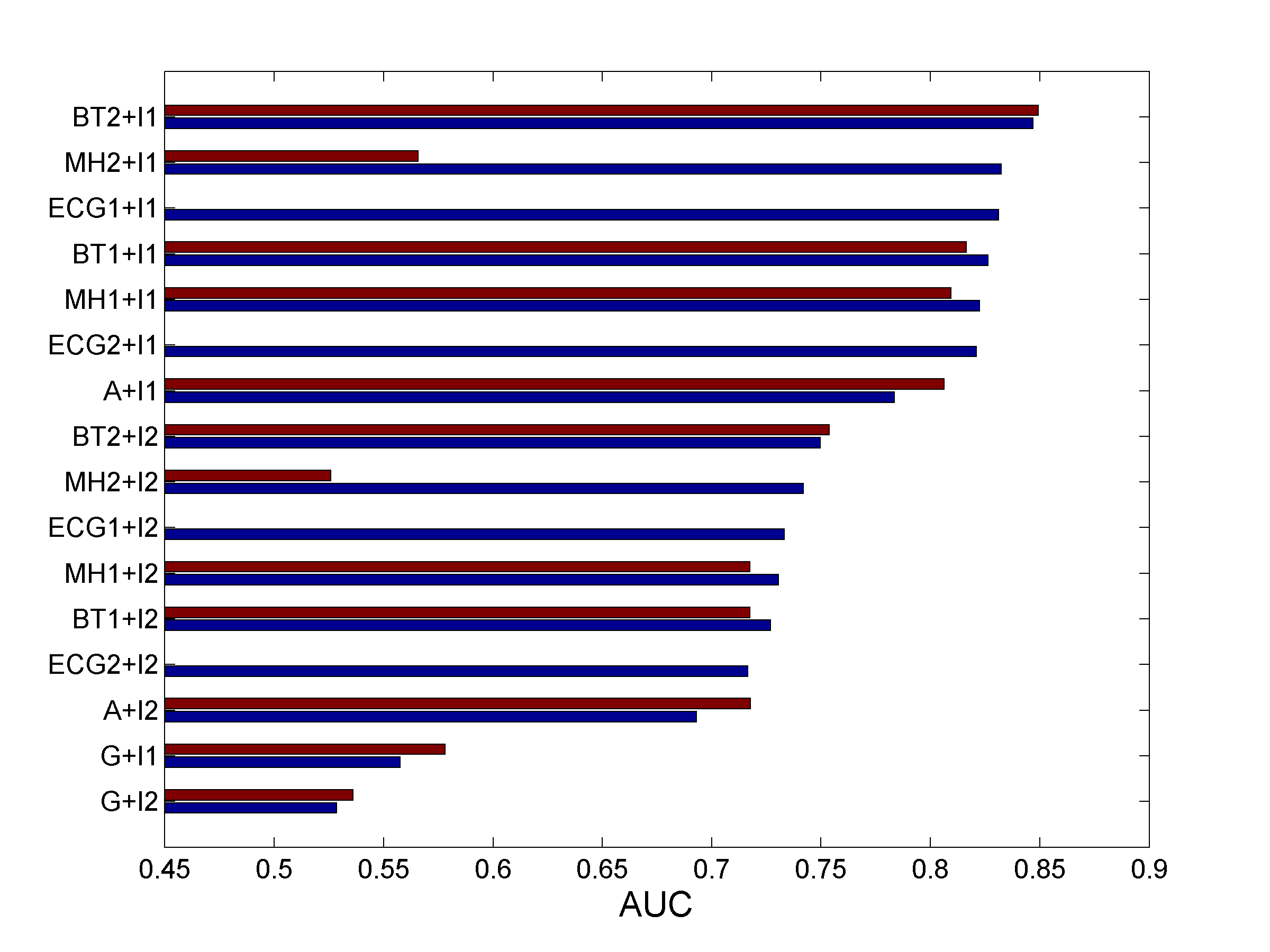

Supplement: Additional file 3: Figure S2 — The performance of individual features in cross validation. Displayed are individual feature AUC scores for the USA data (Red) and ISR data (blue). The abbreviated feature combinations include: ICD hierarchy-based similarity (I1), ICD empirical similarity (I2), Age (A), Gender (G), blood tests- average difference (BT1), blood tests-difference between extremes (BT2), ECG tests- average difference (ECG1), ECG tests-difference between extremes (ECG2), medical history (MH1) and medical history – empirical ICD similarity based (MH2). [file 1741-7015-11-194-S3.tiff]

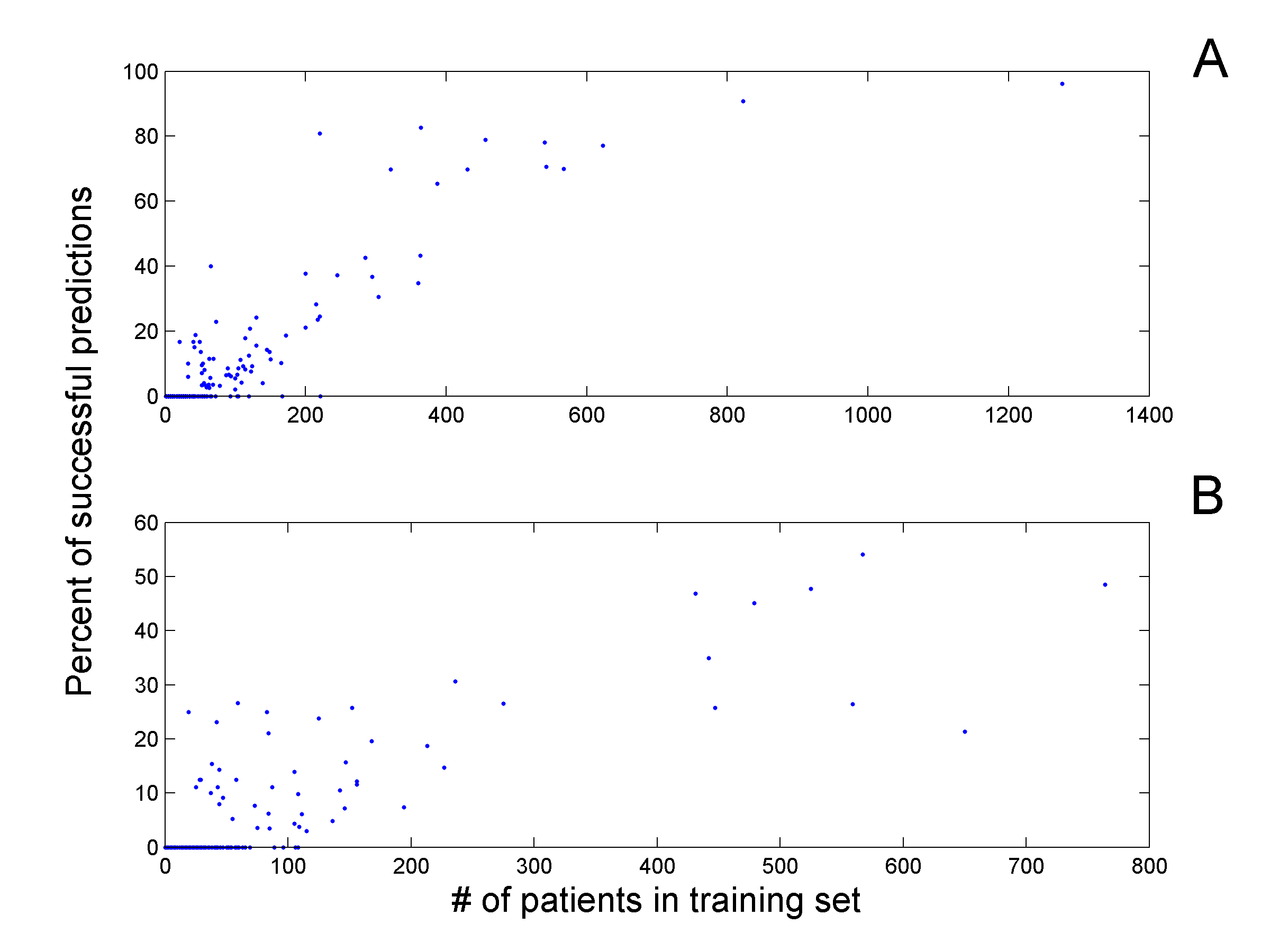

Supplement: Additional file 4: Figure S3 — The precision in predicting ICD codes as a function of the number of patients in the training set for the USA (A) and ISR (B) datasets. [file 1741-7015-11-194-S4.tiff]

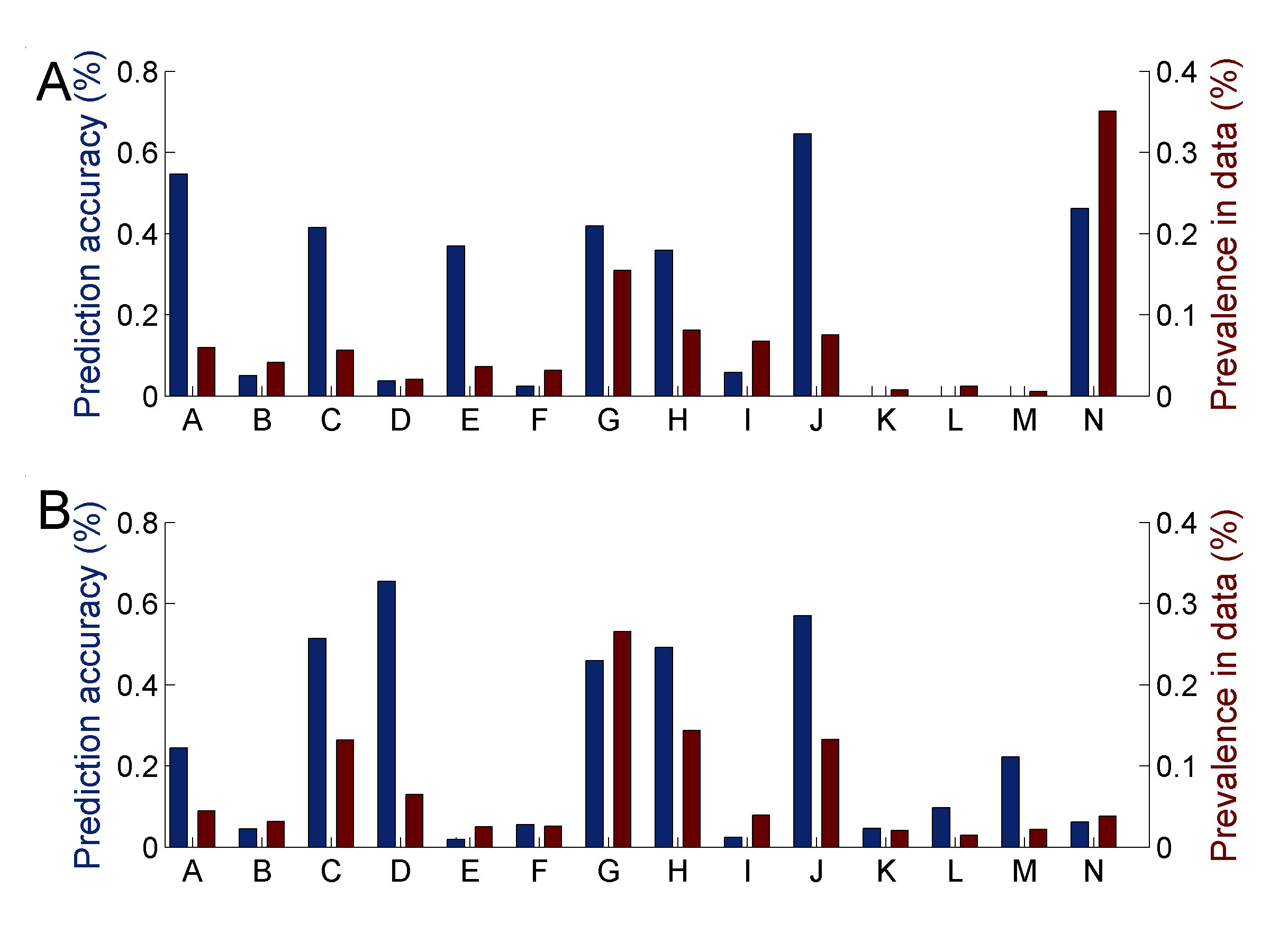

Supplement: Additional file 6: Figure S4 — Prediction precision for ICD level 1 categories. Precision values (blue) and relative prevalence (red) are displayed for the USA (A) and ISR (B) datasets across ICD level 1 categories: Infectious And Parasitic Diseases (A), Neoplasms (B), Endocrine, Nutritional And Metabolic Diseases, And Immunity Disorders (C), Diseases Of The Blood And Blood-Forming Organs (D), Mental Disorders (E), Diseases Of The Nervous System And Sense Organs (F), Diseases Of The Circulatory System (G), Diseases Of The Respiratory System (H), Diseases Of The Digestive System (I), Diseases Of The Genitourinary System (J), Diseases Of The Skin And Subcutaneous Tissue (K), Diseases Of The Musculoskeletal System And Connective Tissue (L), Supplementary Classification Of Factors Influencing Health Status And Contact With Health Services (M) and Classification Of Procedures (N). [file 1741-7015-11-194-S6.tiff]
