# Supplementary material for: A method for inferring medical diagnoses from patient similarities
Source: BMC Med. 2013 Sep 2;11:194. doi: 10.1186/1741-7015-11-194 (PMC3844462; doi:10.1186/1741-7015-11-194)
Supplement: Additional file 5: Table S1 — Easy to predict ICD codes. All p-values are FDR corrected. [file 1741-7015-11-194-S5.pdf]

USA dataset
All p-valus are FDR corrected (FDR of 0.05)

ICD code	ICD description	p-value
038	Septicemia	0
205	Myeloid leukemia	0
250	Diabetes mellitus	0
276	Disorders of fluid, electrolyte, and acid-base balance	0
291	Alcohol-induced mental disorders	0
295	Schizophrenic disorders	0
296	Episodic mood disorders	0
403	Hypertensive chronic kidney disease	0
425	Cardiomyopathy	0
427	Cardiac dysrhythmias	0
428	Heart failure	0
572	Liver abscess and sequelae of chronic liver disease	0
584	Acute kidney failure	0
585	Chronic kidney disease (CKD)	0
39 (procedural)	Other operations on vessels	0
99 (procedural)	Other nonoperative procedures	0
202	Other malignant neoplasms of lymphoid and histiocytic tissue	1.E-05
55 (procedural)	Operations on kidney	4.E-05
54 (procedural)	Other operations on abdominal region	5.E-05
37 (procedural)	Other operations on heart and pericardium	2.E-04
00 (procedural)	Procedures and interventions, Not Elsewhere Classified	2.E-04
277	Other and unspecified disorders of metabolism	2.E-04
486	Pneumonia, organism unspecified	2.E-04
204	Lymphoid leukemia	2.E-04
96 (procedural)	Nonoperative intubation and irrigation	4.E-04
197	Secondary malignant neoplasm of respiratory and digestive systems	4.E-04
491	Chronic bronchitis	6.E-04
253	Disorders of the pituitary gland and its hypothalamic control	6.E-04
288	Diseases of white blood cells	8.E-04
94 (procedural)	Procedures related to the psyche	9.E-04
38 (procedural)	Incision, excision, and occlusion of vessels	1.E-03
305	Nondependent abuse of drugs	2.E-03
599	Other disorders of urethra and urinary tract	2.E-03

Israel dataset
All p-valus are FDR corrected (FDR of 0.05)

ICD code	ICD description	p-value
285	Other and unspecified anemias	0
414	Other forms of chronic ischemic heart disease	0
V42	Organ or tissue replaced by transplant	1.00E-05
275	Disorders of mineral metabolism	6.00E-05
496	Chronic airway obstruction, not elsewhere classified	0.0001
599	Other disorders of urethra and urinary tract	0.0002
491	Chronic bronchitis	0.0003
486	Pneumonia, organism unspecified	0.0006
401	Essential hypertension	0.001
584	Acute kidney failure	0.002
250	Diabetes mellitus	0.003
99 (Procedural)	Other nonoperative procedures	0.004
345	Epilepsy and recurrent seizures	0.004
280	Iron deficiency anemias	0.004
288	Diseases of white blood cells	0.006
V45	Other postprocedural states	0.01
284	Aplastic anemia and other bone marrow failure syndromes	0.01
